# A Plea for Early Operation in Strangulated Hernia

**Published:** 1899-01

**Authors:** William Perrin Nicolson

**Affiliations:** Atlanta, Ga.


### Southern Medical Record.

A MONTHLY JOURNAL OF MEDICINE AND SURGERY.

VOL. XXIX. ATLANTA, GA., JANUARY, 1899. NO. 1.

### Original Articles.

### A PLEA FOR EARLY OPERATION IN STRANGULATED HERNIA.

By WILLIAM PERRIN NICOLSON, M.D., Atlanta, Ga.

In probably no surgical affection does the element of time exert so conspicuous an influence upon the termination of the disease as in strangulated hernia. A little reflection will show that the pathological condition renders it of necessity so. Here we have a delicate tissue, freely supplied with vessels and nerves, constricted to such an extent as to practically completely interrupt its circulation, while at the same time the functions of the alimentary canal are suspended. In addition to this, the pressure upon the nerves produces intense pain, with exceedingly severe nervous depression and shock. Further add to this the uncontrollable vomiting resulting from the interference with the functions of the intestine, and you have a picture that presents nothing but a gloomy outlook. In the face of such a picture we ask, what is the duty of the attending physician? It is clearly to use all possible haste to relieve the constriction, as the danger to the life of the tissues we all know is in direct proportion to the length of time the interruption of the circulation continues. This is in a large number of cases accomplished by simple taxis, especially when the strangulation is very recent. It is not my purpose in this paper to enter into the details of the application of taxis. My only advice would be, do not use it *too long*. Should a careful effort fail to effect reduction, it is the duty of the physician to resort to an anesthetic. Fortunately a large number of hernias, before rebellious, will yield promptly to this and be restored to the abdomen. But let us presume that taxis, carefully applied under the influence of an anesthetic, has failed to reduce the hernia, what is to be done? Many will answer: "Give opium," "Apply ice to the tumor," "Give warm bath," "Let patient rest and try taxis again." Many different replies of such a nature, according to individual opinion. My reply would be, based upon surgical principles, *operate*. At this stage the attending physician, if unwilling to operate, should call surgical counsel while it is "yet day" from the patient's standpoint. It is true that the turning-point of the case for weal or for woe hangs in the balance, and every hour counts heavily against him. This is the golden opportunity. The reasons that urge operative procedure are:

First, the operation *per se* is devoid of danger. In the past operations for strangulated hernia have presented a high mortality, which is readily accounted for in the present light of surgery. Since it involved in most cases opening the peritoneum it was adopted as a last resort, when the patient was reduced to a condition unfit for operative measures of any character. Perhaps the intestine was found gangrenous, or, if not, the patient died of peritonitis, or shock. To-day the picture is entirely changed, and I believe the facts will bear me out in the statement, that the operation performed early, before grave changes have taken place in the gut, or the patient has been exhausted by delay, is devoid of danger. It can not be said to be an operation involving life if cleanly and properly made.

The danger of operation is, in my opinion, far overbalanced by that of returning a piece of intestine into the abdomen when we have no means of knowing its condition. The widest difference prevails in the time required for destructive changes to take place in the strangulated gut. In some instances a strangulation of forty-eight hours will not produce as serious results as one of twelve, the difference arising from the degree of obstruction to the circulation. Hence the return of a hernia by taxis after some duration may be the putting back into the abdomen of a paralyzed or sloughing gut. I recall an instance when a small hernia in a woman was for some time mistaken for an inflamed lymphatic gland until the symptoms of obstruction of the bowels surpervened. As the condition was not very urgent a conservative course was adopted, awaiting the necessity for operation. Finally under taxis the tumor disappeared, but some hours after, the patient, upon rising in bed, fell back in collapse and died within a few hours. Here was evidently a return of a gangrenous gut which subsequently ruptured. An operation that gave an opportunity for inspecting the intestine would have averted this calamity.

For the reason of the value of inspection I would especially deprecate the practice formerly advocated of attempting to reduce the hernia in operative cases without opening the sac.

This was recommended to avoid opening the peritoneunm, no longer a danger in the operation. At the present day an operator who returned a hernia without opening the sac and inspecting the gut would fall far short of his duty. There is nothing in this, or any other surgical work, like *seeing*.

*Lastly*, the opportunity for making a radical cure of the hernia should weigh as a factor in determining operation. In cases where the latter stages have been reached, and the patient is in precarious condition, only the saving of life is to be considered, but in those in whom the operation is made before exhaustion has taken place, there is always an opportunity for curing the diseased condition and ridding the patient of future danger. This, in my opinion, is a factor of great weight in determining upon early operation.

---

### RADIOGRAPHY IN WARFARE.

In the January issue of the *Photogram* an extremely interesting and practical paper appears on this subject, by Dr. J. Hall Edwards. In order to test the practicability of using the X-rays in the field, Dr. Edwards arranged with Surgeon-Major Luke Greer for some special manœuvres at Ward's End (a suburb of Birmingham). These manœuvres were carried out more particularly to disprove the statement of the Under-Secretary for War, that Rontgen apparatus could not be used on the battle-field until the transport difficulties had been overcome. We are pleased to understand that "these were so successful as to prove beyond the shadow of a doubt that the difficulties were more imaginary than real." Dr. Edwards further states that "the experiments were an undoubted success, and tend to prove that not only can the necessary apparatus be transported with perfect ease, but that the actual work is not fraught with insurmountable difficulties. The chemicals for development, fixing, etc., were carried in tubes in the solid state. The sensitive films were ready packed in light-tight envelopes, the only necessary which could not be carried for any distance being a supply of water." It is to be hoped that the chiefs of the Army Medical Staff will see that similar manœuvres are frequently carried out.—*The Therapest.*

